# Cognitive behavioral therapy in the treatment of social phobia

**DOI:** 10.4103/0972-6748.57863

**Published:** 2009

**Authors:** Richa Priyamvada, Sapna Kumari, Jai Prakash, Suprakash Chaudhury

**Affiliations:** Department of Clinical Psychology, RINPAS, Kanke, Ranchi-834006, Jharkhand, India; 1 Department of Psychiatric Social Work, RINPAS, Kanke, Ranchi-834006, Jharkhand, India; 2 Department of Psychiatry, RINPAS, Kanke, Ranchi-834006, Jharkhand, India

**Keywords:** Cognitive behavior therapy, Social phobia, Psychotherapy

## Abstract

Cognitive behavior therapy is probably the most well-known and the most practiced form of modern psychotherapy and has been integrated into highly structured package for the treatment of patients suffering from social phobia. The present case study is an attempt to provide therapeutic intervention program to a 27-year-old, unmarried Christian man suffering from social phobia. The patient was treated by using cognitive behavioral techniques. After 17 sessions of therapeutic intervention program, significant improvement was found. He was under follow-up for a period of 6 months and recovered to the premorbid level of functioning.

Social phobia consists of a marked and persistent fear of encountering other people, usually in small groups; or doing certain acts in a public place, like eating in public toilets, public speaking or encounters with persons of the opposite sex. Affected individuals fear that they will be evaluated negatively or that they will act in a manner that resulting in their humiliation or embarrassment whenever they are expected to go into the phobic situations; they develop severe anticipatory anxiety. They utilize various excuses to avoid phobic situations. This avoidance usually affects their lives quite adversely. Many of these patients exhibit psychological symptoms of poor self confidence, show anxiety on trifles and may be very conscious of some physical or psychological defect in them; as a result, they may develop secondary depression. Exposure to social situations can produce physical symptoms such as sweating, blushing, muscle tension, pounding heart, dry mouth, nausea, urgency of masturbation, shaky voice or trembling. Social phobia is the third most common mental disorder in adults worldwide, with a lifetime prevalence of at least 5% (depending on the threshold for distress and impairment). There is an equal gender ratio in treatment settings; but in catchment area surveys, there is a female preponderance of 3:2. Affected individuals are more likely to be unmarried and have a low socioeconomic status. Although common, social phobia is often not diagnosed or effectively treated. There have however been a number of developments in our understanding and treatment of social phobia over the past decade. Cognitive and behavioral interventions for social phobia appear to be more effective than wait-list controls and supportive therapy. Cognitive behavioral treatment involving cognitive restructuring plus exposure appears to be an effective treatment and exhibits a larger effect than either exposure or social skills training or cognitive restructuring alone. The sessions of CBT for social phobia are devoted to training clients in the basic tenets of cognitive therapy, especially the link between faulty assumptions or irrational thinking about social situations and anxiety experienced in those situations (Albano & DiBartolo, 2007; David, 2003; Leichsenring *et al*., 2009).

## CASE REPORT

The patient was a 27-year-old man suffering from social phobia, youngest in his family, unmarried, graduate, having average socioeconomic status and hailing from Jharkhand state, India. The patient came to RINPAS OPD with complaints of fearfulness in crowd, sweating, low confidence, negative thoughts, decreased interaction and inferiority complex. The duration of illness was 5 to 6 years. The patient had difficultly in carrying out his daily routine; consequently, he came for treatment. It was revealed from his history that he was fearful as compared to other persons of his age; from childhood, his mother was overprotective about him. His father was dominating and did not listen to anyone in the family; the patient was very scared of his father. Due to fearfulness, he remained dependent on others for the completion of his simple tasks. Gradually he started avoiding gatherings and crowd and did not go out of home. He felt difficulty in interacting with unknown people and even in opening up with people with whom he was familiar. He was unable to talk with them in a crowd. He thought that he did not have a good pattern of behavior and could not behave like other people. Although he put in efforts to behave normally, yet he sweated a lot during public interaction. Whenever he went out in social gatherings, he thought people were avoiding him, and he felt inferior or disapproved. His self-esteem decreased gradually as he could not take initiative in any activity. Negative ideas also developed in his mind — that he would never flourish; he would not be successful; he would not be able to behave like other people in society. Behavioral analysis was done with regard to antecedent frequency, duration, intensity and motivation of the patient in order to target behavior. Assessment regarding family interaction system, available support system and perceived support system, as well as behavior of other significant persons towards the patient, was done systematically.

## THERAPEUTIC PROCESS

### Assessment of the problem

At first, rapport was established with the patient and then clinical interview was conducted, in view of the fact that the patient was suffering since the last 5 to 6 years. Due to fear in social gatherings, the patient was unable to interact with unknown people. He had lost his confidence and was unable to perform his work efficiently. Whenever he went to new places, he started sweating. He also suffered from inferiority complex and had lost his interest in work. He was unable to maintain his daily routine as he was lethargic. Most of the time, he was worried about his problems and was unable to overcome this feeling of worry. This severely disturbed his social functioning, and he developed depressive features and poor self-esteem.

### Tools

The following tools were used for assessing the patient.

#### Beck depression inventory (Beck & Steer, 1990)

To assess the severity of depression, Beck Depression Inventory (BDI) was administered. Assessment revealed mild level of depression in the patient. Lack of satisfaction, sense of failure, indecisiveness, sleep disturbance, hopelessness and guilt feelings were the main features.

#### Social phobia inventory (Liebowitz, 2002)

The SPIN is a self-rated questionnaire that measures the 3 commonly seen types of social anxiety disorders: f0 ear; phobic avoidance; and autonomic symptoms such as blushing, sweating and trembling.

### Objectives of the intervention

After assessment of the problem, the intervention package focused on the following:

- To motivate the patient for therapy.
- To prepare him to deal with and face phobic situations he avoided due to anxiety.
- To reduce his anxiety.
- To reduce inferiority complex and increase self-esteem.
- To modify his negative thoughts.

The therapeutic package consisted of the following intervention techniques

1. Psycho-education
2. Jacobson’s progressive muscular relaxation technique
3. Systematic desensitization
4. Exposure and response prevention technique
5. Cognitive restructuring

Seventeen sessions of cognitive behavior therapy were conducted over a period of 15 weeks to achieve the goal. Each session lasted for 1 hour to 1½ hours. First of all, the patient was explained the basic nature and purpose of cognitive therapy; also, the significance of this collaborative approach was discussed in detail. Then, a therapeutic intervention strategy was planned, and the following behavioral and cognitive techniques were implemented. The therapeutic intervention program started with psycho-education. Although the patient had partial insight about his problem, as he recognized few of his symptoms as a part of illness, yet to enhance the insight adequately, proper counseling was done. The patient was explained about the nature, symptomatology, causative factors, course and maintaining factors of the illness. In the next session, the role of medication and psychotherapy as a process of treatment for the purpose of recovery from illness was explained to him. In the next session, for reducing anxiety, training in Jacobson’s progressive muscular relaxation technique was given to the patient. It started with breathing exercise, and then the patient underwent Jacobson’s progressive muscular relaxation process. Training was given in several sessions, and he was persuaded to practice the process at home. After following this process, his anxiety gradually reduced, which was concluded from the fact that the patient himself confirmed that he was then unable to deal with fewer situations. After discussion with the patient in the next session, systematic desensitization was done, which involves gradual exposure to phobic stimulus along hierarchy of increasing intensity, and was continued until the patient was habituated with the situation and avoidance response was extinguished. Relaxation technique was also used before situational exposure. The procedure of exposure and response prevention was adopted to evoke anxiety in the patient, who was advised to attend social gatherings and present a speech in front of few people. Also, his friends were requested to monitor the exposure and response prevention and keep an eye on noticeable changes in his behavior. It was noticed that systematic desensitization and exposure with response prevention helped reduce his anxiety level. In subsequent sessions, in order to modify his negative thoughts and faulty cognition, cognitive restructuring was done, in which attempts were made to restructute all the negative and wrong beliefs he had developed from his childhood. He was taught how to challenge the negative thoughts in a rational, objective and analytical manner by himself.

Marked improvement was noticed after 17 sessions of therapeutic intervention program. The level of anxiety and guilt feeling decreased. His self-esteem increased and he was able to attend social gatherings; also, his negative thoughts about himself were modified. This helped in the recovery of his illness. The patient expanded the activities of the institution where he worked as part of an NGO to other cities and was able to attend various social gatherings. At the end of 6 months, follow-up was done. There was significant improvement, which ultimately led the patient to maintain a normal daily routine.

## DISCUSSION

**Figure 1:**
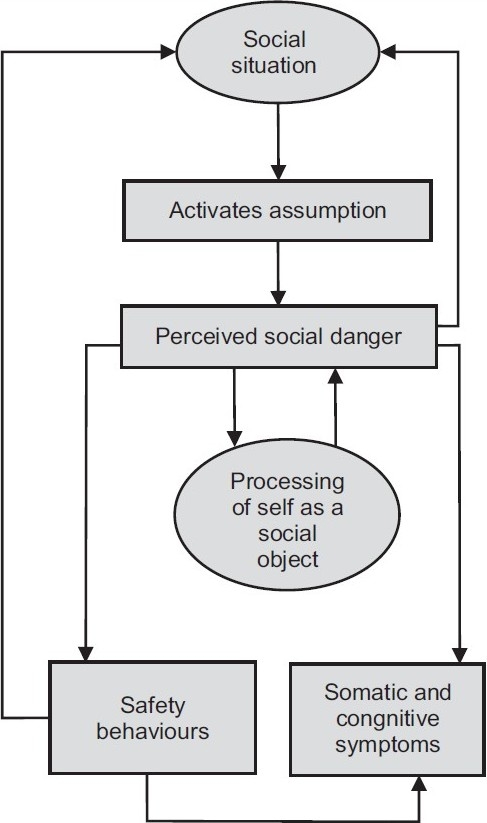
A cognitive model of social phobia

Clark & Wells (1995) and Clark (2001) have developed a cognitive model for the management of social phobia [Figure 1]. The aim of the model was to answer the question of why the fears of someone with social phobia are maintained despite frequent exposure to social or public situations and nonoccurrence of the feared catastrophes. The model suggests that when patients enter a social situation, certain rules (e.g., “I must always appear witty and intelligent.”), assumptions (e.g., “If a woman really gets to know me, she will think I am worthless.”) or unconditional beliefs (e.g., “I am weird and boring.”) are activated. When individuals believe that they are in danger of negative evaluation, an attentional shift occurs towards detailed self-observation and monitoring of sensations and images. Socially anxious individuals thus use internal information to infer how others are evaluating them. [In Figure 1, this is ‘processing of self as a social object.’] The internal information is associated with feelings of anxiousness, and vivid or distorted images are imagined from an observer’s perspective. These images are mostly visual, but they might also include bodily sensations and auditory or olfactory perspectives. This is not, of course, what an observer actually ‘sees.’ Recurrent images can be elicited by asking patients to recall a social situation associated with extreme anxiety. The images are usually linked to early memories. The therapist asks the patient whether he or she remembers first having the experience encapsulated in the recurrent image and to recall the sensory features and meaning that this had. For example, someone who had an image of being fat remembered being teased during adolescence, which resulted at the time in feelings of humiliation and rejection. A second factor that maintains symptoms of social phobia is safety behaviors. These are actions taken in feared situations in order to prevent feared catastrophes. Safety behaviors in social phobia include using alcohol; avoiding eye contact; gripping a glass too tightly; excessive rehearsing of a presentation; reluctance to reveal personal information; and asking many questions. Safety behaviors are often problematic: t0 hey prevent disconfirmation of the feared catastrophe; they can heighten self-focused attention and monitoring to determine if the behavior is ‘working’; they increase the feared symptoms (e.g., keeping arms close to the body to stop others seeing one sweat will increase sweating); they have an effect on others (e.g., the individual may appear cold and unfriendly, so that a feared catastrophe becomes a self-fulfilling prophecy); and they can draw attention to feared symptoms (e.g., speaking quietly and slowly will lead others to focus on the individual even more). It is hypothesized that a third factor that maintains symptoms of social phobia is anticipatory and post-event processing. Such processing focuses on the feelings and constructed images of the self in the event and leads to selective retrieval of past failures.

The results of the present case study are in agreement with those of earlier studies that indicate the significance of CBT in the treatment of patients suffering from social phobia (Ponniah & Hollon, 2008). Psycho-education proved to be very useful in understanding the dynamics of the patient problems, as well as to enable the patient to proceed in positive direction with the help of emotional support. There was decrease in anxiety and distress. Anxiety improved with the practice of Jacobson’s progressive muscular relaxation technique. Similar results have been reported by David (2004). Exposure techniques involve repeatedly facing previously avoided situations in a graded manner until habituation occurs. Cognitive restructuring helped in modifying negative automatic thoughts, which in turn helped in improving the patient’s self-esteem and changing the patient’s perception and way of thinking about the world and himself as well. Cognitive treatment is useful in restructuring and modifying the patient’s negative cognitive beliefs towards himself and others. The emphasis is on shifting the focus of attention, dropping safety behaviors, processing the situation and evaluating what was predicted against what actually happened.

## CONCLUSION

The case report highlights the fact that combination of cognitive, emotional and behavioral approaches is effective and is the initial choice of treatment for social phobia.
